# Use of Extracorporeal Photopheresis Treatment Over Time (2001–2023) in Alberta, Canada: A Retrospective Cohort Study

**DOI:** 10.1002/hsr2.71373

**Published:** 2025-10-16

**Authors:** Louis Girard, Sylvia Aponte‐Hao, Jason R. Randall, Karen J. B. Martins, Huong Luu, Khanh Vu, Tyler Williamson, Scott W. Klarenbach

**Affiliations:** ^1^ Department of Medicine, Cumming School of Medicine University of Calgary Calgary Alberta Canada; ^2^ Data and Research Services Alberta SPOR SUPPORT Unit Data Platform Calgary Alberta Canada; ^3^ Centre for Health Informatics, Cumming School of Medicine University of Calgary Calgary Alberta Canada; ^4^ Real World Evidence Unit, Faculty of Medicine and Dentistry University of Alberta Edmonton Alberta Canada; ^5^ Alberta Children's Hospital Research Institute University of Calgary Alberta Canada; ^6^ Libin Cardiovascular Institute University of Calgary Alberta Canada; ^7^ O'Brien Institute for Public Health University of Calgary Alberta Canada; ^8^ Department of Community Health Sciences, Cumming School of Medicine University of Calgary Alberta Canada; ^9^ Department of Medicine, Faculty of Medicine and Dentistry University of Alberta Edmonton Alberta Canada

**Keywords:** administrative data, cutaneous T‐cell lymphoma, graft‐versus‐host disease, photochemotherapy, photoimmunotherapy, photopheresis

## 1 Introduction

Extracorporeal photopheresis (ECP) is an immunomodulatory procedure involving the exposure of isolated lymphocytes from peripheral blood to 8‐methoxypsoralen and ultraviolet A radiation, followed by their re‐infusion into the patient [1, 2]. Although the precise mechanism of action is not fully understood, the treatment effect may be mediated by lymphocyte apoptosis and immunomodulation [3, 4]. ECP was initially approved by Health Canada in 1999 for the treatment of cutaneous T‐cell lymphoma (CTCL), a rare type of cancer originating in white blood cells (ECP strongly recommended as first line therapy by the American Society for Apheresis [5]), and subsequently approved for systemic sclerosis, a rare connective tissue disorder (ECP weakly recommended; individual decision‐making suggested [5]). In addition to these approved indications, ECP is routinely used as an off‐label treatment for graft‐versus‐host disease (GVHD), a systemic disorder that occurs when donated immune cells recognize the host as foreign and attack them (ECP strongly recommended as second line therapy [5]); there is emerging evidence for ECP treatment in other cancers and immunologically mediated disorders [5]. Although ECP has been in use for over three decades, there is limited information on its use in Canada [6]. The objective of this study was to describe the characteristics of those who received ECP treatment in Alberta, Canada.

## 2 Methods

Ethics approval for this retrospective, observational, population‐based cohort study was received from the University of Alberta Research Ethics Board (Pro00123337); informed consent was waived. Data custodian approvals were received. This study was reported according to the Strengthening the Reporting of Observational Studies in Epidemiology guidelines. In Alberta, the fourth most populous province in Canada (3.1 to 4.7 million people between 2001 and 2023), publicly funded healthcare is administered under the Alberta Health Care Insurance Plan (AHCIP), of which over 99% of Albertans participate [7, 8]. A person‐level data extract of all individuals in the province who received ECP between January 2001 and March 2023 (observation period) was obtained from the Calgary Apheresis clinical database (includes all photopheresis treatments at the Foothills Medical Centre [for those aged ≥ 18 years] and the Alberta Children's Hospital [for those aged < 18 years]; these two centers were the only locations where ECP treatment occurred in Alberta during the study period) and linked to the Population Registry (contains demographic information for all Albertans with AHCIP coverage) using a unique individual identifier (Personal Health Number). Study measures included the total number of individuals who received ECP, the indication for treatment, and the number of treatments received, along with the demographic characteristics of age, sex, and urban/rural residence (determined by the second digit of the postal code) on the first date of ECP treatment during the observation period. Descriptive statistics were reported as counts and percentages, means and standard deviations (SD), or medians and interquartile ranges (IQR) where applicable. In accordance with data custodian privacy standards, outcomes with 1–9 individuals were reported as < 10 or censored in a manner that prevented calculation of the small cell number. Analysis was performed using R (version 4.4.0; R Development Core Team) [9].

## 3 Results

The UVAR‐XTS (2001‐2013) and the Cellex (2013 onwards) ECP closed systems were used (Therakos, Mallinckrodt Pharmaceuticals, Ireland). A total of 189 individuals were identified as having received ECP for a total of 9,369 treatments (median of 28 [IQR 12–60] treatments each) during the observation period; 132 individuals were linked to the Provincial Registry and had demographic information (Table 1). Mean age at initiation of ECP was 51 (SD 17) years (< 10 were aged < 18 years), and 52.3% (*n* = 69 of 132) were male (Table 1). A total of 86.4% (*n* = 114 of 132) lived in an urban area; 56.1% (*n* = 74 of 132) lived in Calgary (Table 1).

**Table 1:** Characteristics of those who received extracorporeal photopheresis.

**ECP‐related characteristics**		**Demographic characteristics**	
Characteristics	Total cohort (*n* = 189)	Characteristics	Linked group (*n* = 132)
First ECP treatment		Age, years	
2001–2005	41 (21.7%)	Mean (SD)	51 (17)
2006–2010	43 (22.8%)	Median (IQR)	56 (42–62)
2011–2015	66 (34.9%)	Missing	6
2016–2020	40 (21.2%)	Sex	
2021–2023	28 (14.8%)	Male	69 (52.3%)
Indication		Female	63 (47.7%)
GVHD	117 (61.9%)	Missing	0
CTCL	45 (23.8%)	Residence	
All others	27 (14.3%)	Urban/rural	
Number of ECP treatments		Urban	114 (86.4%)
Total	9,369	Rural	18 (13.6%)
Overall, per person		Missing	0
Mean (SD)	50 (64)	AHS health zone	
Median (IQR)	28 (12–60)	Calgary	74 (56.1%)
GVHD, per person		Edmonton	27 (20.6%)
Mean (SD)	56 (73)	Central	10‐18 ( ~ 10.6%)
Median (IQR)	32 (12–74)	South	12 (9.2%)
CTCL, per person		North	< 10 ( ~ 3.8%)
Mean (SD)	41 (43)	Missing	0
Median (IQR)	30 (15–48)		

Abbreviations: AHS, Alberta Health Services; CTCL, cutaneous T‐cell lymphoma; ECP, extracorporeal photopheresis; GVHD, graft‐versus‐host disease; IQR, interquartile range; SD, standard deviation.

**Figure 1:**
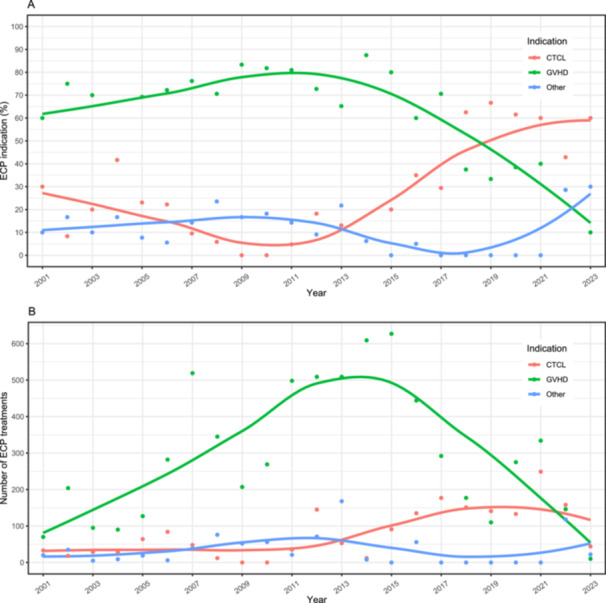
Trends in extracorporeal photopheresis (ECP) use by indication between 2001 and 2023 presented according to the annual (A) proportion who received ECP, and (B) number of ECP treatment runs. The indications tracked are cutaneous T‐cell lymphoma (CTCL, red), graft‐versus‐host disease (GVHD, green), and “Other” (blue). (A) GVHD was the predominant indication, peaking in approximately 2012 before steadily declining. CTCL steadily increased from 2012 onwards and surpassed GVHD in 2018. The “Other” category was relatively low initially but markedly increased after 2019/2021, surpassing GVHD by 2023. (B) The number of ECP treatments were predominantly for GVHD, peaking in 2015 and then declining below CTCL and “Other” in 2023. The number of ECP treatments for CTCL increased from 2014 onwards, and “Other” was generally low.

The number of individuals who received ECP increased from 2001 to 2015 (41 received treatment in 2001–2005; 43 received treatment in 2006–2010; 66 received treatment in 2011–2015), and decreased thereafter (40 received treatment in 2016–2020; 28 received treatment in 2021–2023) (Table 1). Overall, the majority of individuals received ECP for GVHD (61.9%, *n* = 117 of 189; median of 32 [IQR 12–74] treatments each), followed by CTCL (23.8%, *n* = 45 of 189; median of 30 [IQR 15–48] treatments each); all other indications for which ECP was received (14.3%; *n* = 27 of 189) included systemic sclerosis, solid organ transplant rejection, Crohn's disease, multiple sclerosis, dermatomyositis, hypereosiniphilic syndrome, lymphoma, and leukocytoclastic vasculitis (< 10 each per health condition) (Table 1). Those who received ECP for specific health conditions changed over the observation period (Figure 1A). The majority received ECP for GVHD from 2001 to 2017. From approximately 2012 onwards, the proportion who received ECP for GVHD decreased, and the proportion who received ECP for CTCL and all other health conditions increased; by 2023, the majority who received ECP were living with CTCL, followed by all other indications collectively, and then GVHD. The total annual number of ECP treatments presented according to indication is shown in Figure 1B.

## 4 Discussion

In this retrospective, observational, population‐based cohort study, the characteristics of 189 individuals who received ECP treatment between 2001 and 2023 in Alberta, Canada were described. ECP has been reported to be underutilised due in part to limited access and time commitment [10]. In Canada, ECP programs are offered in six cities (Calgary, Montreal, Toronto, Saskatoon, Vancouver, and Winnipeg), with three sites offering treatment to the pediatric population. In this study, a larger proportion of individuals who received ECP resided in Calgary than would be anticipated—56.1% lived in Calgary where 30.7% of the Alberta population resides [11]—likely reflecting geographical barriers influencing care management decisions for ECP treatment in Alberta.

GVHD was the predominant indication for ECP treatment in this study until approximately 2012, after which its use declined, likely reflecting changes in the therapeutic landscape for GVHD. Immunosuppression with corticosteroids forms the basis of first‐line treatment in acute and chronic GVHD [12, 13]. Until recently, a number of different non‐standard second‐line treatments were used for steroid‐resistant or steroid‐dependent GVHD, including ECP. The introduction of ruxolitinib, a selective Janus kinase (JAK) 1/2 inhibitor that became available in Canada in 2012, has shifted treatment recommendations—the 2024 Cell Therapy Transplant Canada guidelines recommend ruxolitinib as the second‐line treatment for steroid‐resistant or steroid‐dependent GVHD, with ECP recommended as a third‐line option or beyond [13].

Strengths of this study include the use of population level data. This study is also subject to limitations that should be taken into consideration when interpreting results. Subtypes of health conditions for which ECP was received were not consistently delineated in the Calgary Apheresis clinical database, and therefore not reported. Linkage to the Population Registry (contains demographic information) was not available for all individuals who received ECP. Results may not necessarily be generalizable to other provinces.

## 5 Conclusions

This study provides important information on the characteristics of those who received ECP treatment in Alberta, Canada during a 22 year period (2001–2023). Findings highlight that access to ECP treatment was likely not equitable; identifying and overcoming barriers to optimal clinical care for those who stand to benefit from this treatment is needed. The finding that health conditions for which ECP was received changed over time can be used to guide resource allocation in the evolving landscape of ECP treatment and new therapeutics.

## Author Contributions

**Louis Girard:** conceptualization, writing – review and editing. **Sylvia Aponte‐Hao:** conceptualization, methodology, data curation, software, formal analysis, investigation, visualization, writing – review and editing. **Jason R. Randall:** conceptualization, methodology, writing – review and editing, project administration. **Karen J. B. Martins:** conceptualization, writing – original draft, writing – review and editing, project administration. **Huong Luu:** conceptualization, validation, writing – review and editing. **Khanh Vu:** conceptualization, validation, writing – review and editing. **Tyler Williamson:** conceptualization, writing – review and editing. **Scott W. Klarenbach:** conceptualization, writing – review and editing, methodology, supervision, funding acquisition.

## Conflicts of Interest

The author(s) declared the following potential conflicts of interest with respect to the research, authorship, and/or publication of this report: S.A.H., J.R., K.M., H.L., K.V., T.W., and S.K. are members of the Alberta Real World Evidence Consortium (ARWEC) and the Alberta Drug and Therapeutic Evaluation Consortium (ADTEC); these entities (comprised of individuals from the University of Alberta, University of Calgary, and Institutes of Health Economics) conduct research including investigator‐initiated industry‐funded studies (ARWEC) and government‐funded studies (ADTEC). Mallinckrodt Pharmaceuticals is a provider of extracorporeal photopheresis delivery systems, and provided research funding for this study to the University of Alberta, with S.K. as the principal investigator. The other authors declare no conflicts of interest.All authors of this study had complete autonomy over the design and execution of the study, as well as the content and submission of the report. All authors have read and approved the final version of the manuscript. S.K. had full access to all of the data in this study and takes complete responsibility for the integrity of the data and the accuracy of the data analysis.

## Transparency Statement

The lead author Scott W. Klarenbach affirms that this manuscript is an honest, accurate, and transparent account of the study being reported; that no important aspects of the study have been omitted; and that any discrepancies from the study as planned (and, if relevant, registered) have been explained.

## Data Availability

The data that support the findings of this study are available from the Calgary Apheresis clinical database and Alberta Health Services, but restrictions apply to the availability of these data, which were used under license for the current study, and so are not publicly available.
